# Automatic extraction of semantic relations between medical entities: a rule based approach

**DOI:** 10.1186/2041-1480-2-S5-S4

**Published:** 2011-10-06

**Authors:** Asma Ben Abacha, Pierre Zweigenbaum

**Affiliations:** 1 LIMSI, CNRS, Orsay, F-91403, France

## Abstract

**Background:**

Information extraction is a complex task which is necessary to develop high-precision information retrieval tools. In this paper, we present the platform MeTAE (Medical Texts Annotation and Exploration). MeTAE allows (i) to extract and annotate medical entities and relationships from medical texts and (ii) to explore semantically the produced RDF annotations.

**Results:**

Our annotation approach relies on linguistic patterns and domain knowledge and consists in two steps: (i) recognition of medical entities and (ii) identification of the correct semantic relation between each pair of entities. The first step is achieved by an enhanced use of MetaMap which improves the precision obtained by MetaMap by 19.59% in our evaluation. The second step relies on linguistic patterns which are built semi-automatically from a corpus selected according to semantic criteria. We evaluate our system’s ability to identify medical entities of 16 types. We also evaluate the extraction of treatment relations between a treatment (e.g. medication) and a problem (e.g. disease): we obtain 75.72% precision and 60.46% recall.

**Conclusions:**

According to our experiments, using an external sentence segmenter and noun phrase chunker may improve the precision of MetaMap-based medical entity recognition. Our pattern-based relation extraction method obtains good precision and recall w.r.t related works. A more precise comparison with related approaches remains difficult however given the differences in corpora and in the exact nature of the extracted relations. The selection of MEDLINE articles through queries related to known drug-disease pairs enabled us to obtain a more focused corpus of relevant examples of treatment relations than a more general MEDLINE query.

## Introduction

Medical knowledge is growing significantly every year. According to some studies, the volume of this knowledge doubles every five years [1], or even every two years [2]. With large-scale digitisation, several medical search engines went on display, such as PubMed [3] for searching biomedical literature, CISMeF [4], catalog and index of French medical Web sites or Health On the Net [5], a public medical search engine. However, while these search engines have a big contribution in making large volumes of medical knowledge accessible, their users have often to deal with the burden of browsing and filtering the numerous results of their queries in order to find the precise information they were looking for. This point is more crucial for practitioners who may need an immediate answer to their queries during their work.

In this context, we need systems able to respond to users queries with precise answers. Such tools need deep analysis of biomedical documents in order to extract relevant information. At the first level of this information come the medical entities (e.g. diseases, drugs, symptoms). At the second, more complicated level comes the extraction of semantic relationships between these entities.

In this paper, we present our method to extract semantic relations between medical entities, with an empirical study on the “treatment” relation. We first propose an enhanced use of MetaMap [6] to extract medical entities and compare it with the simple application of MetaMap on the same test corpora. To extract occurrences of the target relations, we then design linguistic patterns based on selected sentences from PubMed Central articles. We present a method to obtain such sentences by leveraging UMLS Metathesaurus knowledge and MeSH indexing of PubMed Central. We evaluate entity and relation extraction on a distinct corpus of 580 sentences and obtain promising results. We also present MeTAE, a platform for automatic semantic annotation and exploration of medical texts which incorporates these information extraction components and allows querying the obtained information. We finally discuss our results and conclude on further work.

## Background

MetaMap [6] is a reference tool for medical entity recognition which allows mapping medical text to UMLS concepts. Using MetaMap therefore provides a strong baseline to start with. MetaMap is able to identify most concepts in the titles of articles from MEDLINE [7]. Meystre and Haug [8] obtained good precision and recall measures (resp. 0.753 and 0.892) with an approach based on MetaMap for extracting “medical problems”. However, the use of MetaMap leads to some residual problems at two levels: (i) in the segmentation and the extraction of medical entities: MetaMap considers some general words and some verbs as medical entities (e.g. best, normal, take, reduce) and (ii) in the categorization of medical entities: MetaMap may propose several concepts for the same term as well as several semantic types for the same concept. We address these two issues in our system by performing independent segmentation of the text before giving it to MetaMap, then imposing constraints on the semantic types of concepts it detects. Domain-independent relation extraction has been studied by a wide range of approaches which can be classified in four categories. Statistical approaches based on term frequency and co-occurrence of specific terms [9], machine learning techniques [10], linguistic approaches [11] (e.g. using manually written extraction rules) and hybrid approaches which combine two or more of the preceding methods [12]. In the medical domain, the same strategies can be found but the specificities of the domain led to specialised methods. Cimino and Barnett [13] used linguistic patterns to extract relations from titles of Medline articles. The authors used MeSH headings and co-occurrence of target terms in the title field of a given article to construct relation extraction rules. Khoo et al. [14] focused on extracting causal relations from abstracts of biomedical articles by aligning manually-constructed graph patterns with syntactic dependency trees. Lee et al. [15] used UMLS to identify semantic relations between medical entities. Their first method could extract 68% of the semantic relations in their test corpus but if many relations were possible between the relation arguments no disambiguation was performed. Their second method [16] targeted the precise extraction of “treatment” relations between drugs and diseases. Manually written linguistic patterns were constructed from medical abstracts talking about cancer. Their system reached 84% recall but an overall 48.14% precision. Embarek and Ferret [17] proposed an approach to extract four kinds of relations (*Detect, Treat, Sign* and *Cure*) between five kinds of medical entities. The patterns used were constructed automatically using an alignment algorithm wich maps sentence parts using an edit distance (defined between two sentences) and different word-level clues. SemRep [18], a natural language processing application, targeted the extraction of semantic relationships in biomedical text through a rule-based approach. SemRep [19] obtained a 53% recall and 67% precision in identifying risk factors and biomarkers for diseases asserted in MEDLINE citations. An enhanced version of SemRep [20] was proposed to identify core assertions on pharmacogenomics and obtained an overall 55% recall and 73% precision. Domain-independent relation extraction methods are not directly applicable to the medical domain due to the lack of domain independent markers that may help to recognise medical entities (e.g. capital letters, regular grammatical structure) and to the variety in the expression of domain concepts (e.g. Amoxicillin = amoxycillin = AMOX). To bypass these problems, medical relation extraction approaches often rely on domain knowledge such as the UMLS Metathesaurus and Semantic Network. But the post-use of extracted relations is not always taken into account in the extraction procedure. For instance, if the extracted relations are to be used in keyword querying systems, we should either give priority to recall or give the same priority for recall and precision, while, if the final application is a question answering system for practitioners, priority should be given to the precision of extraction. Medical relation extraction approaches sometimes also do not care about extracting the arguments of a relation (e.g. [16]), or evaluate their approaches by counting relations extracted with only one argument as correct (e.g. [21]), considering that recall is the most important measure. In our context we are interested in medical question answering systems as back-end and give priority to precision, considering the correct extraction of arguments as mandatory to validate the identified relations.

Most relation extraction methods rely on a corpus where example occurrences of the target relations can be found. For instance, given pairs of seed terms which are known to entertain the target relation, semi-supervised methods such as that introduced in [11] collect occurrences of these term pairs in the corpus and use them to build relation patterns. The selection of a relevant corpus is a key point here: for such a method to work, the corpus must contain mentions of the target relationship between these pairs of terms. We propose a method to increase the chances that such mentions are actually found in the selected texts.

## Method

Our annotation method is twofold. In a first step, we extract medical entities from sentences and determine their categories. In a second step, we extract semantic relations between the extracted entities using lexical patterns. In this section we describe our approach for medical entity recognition, relation extraction and patterns construction before presenting our evaluation method.

### Medical entity recognition

By “medical entity”, we refer to an instance of a medical concept such as Disease or Drug. Medical entity recognition consists in: (i) identifying medical entities in the text and (ii) determining their categories. For instance, in the following sentence “ACE inhibitors reduce major cardiovascular disease outcomes in patients with diabetes.”, the medical entity *ACE inhibitors* should be identified as a treatment and the medical entity *cardiovascular disease outcomes* should be identified as a problem.

One of the most important obstacles to identifying medical entities is the high terminological variation in the medical domain (e.g Swine influenza = swine flu = pig flu).

MetaMap [6] deals with this variation by using morphological knowledge found in the UMLS Specialist Lexicon and term variants present in the UMLS Metathesaurus. However, as mentioned in the Background section, some issues must still be addressed. According to empirical observations, the sentence and noun phrase segmentations provided by MetaMap is not as performant as the segmentation provided by other non-specialized tools known in Natural Language Processing. Besides, a disambiguation step is required on the obtained concepts.

To solve these problems, we propose an approach in three points:

1. Split the biomedical texts into sentences and extract noun phrases with non-specialized tools. We use LingPipe [22] and Treetagger-chunker [23] which offer a better segmentation according to empirical observations.

2. Determine medical entities as well as UMLS concepts and semantic types with MetaMap.

3. Filter the obtained medical entities with (i) a list of the most frequent/noticeable errors and (ii) a restriction on the semantic types used by MetaMap in order to keep only semantic types which are sources or targets for the targeted relations (cf. Table 1).

**Table 1:** Examples of categories and corresponding UMLS semantic types

Category	Examples of UMLS Semantic Types
**Problem**	*Anatomical Abnormality, Injury or Poisoning, Disease or Syndrome*
**Treatment**	*Pharmacologic Substance, Therapeutic or Preventive Procedure*
**Test**	*Diagnostic Procedure, Laboratory Procedure*

### Relation extraction

**Figure 1:**
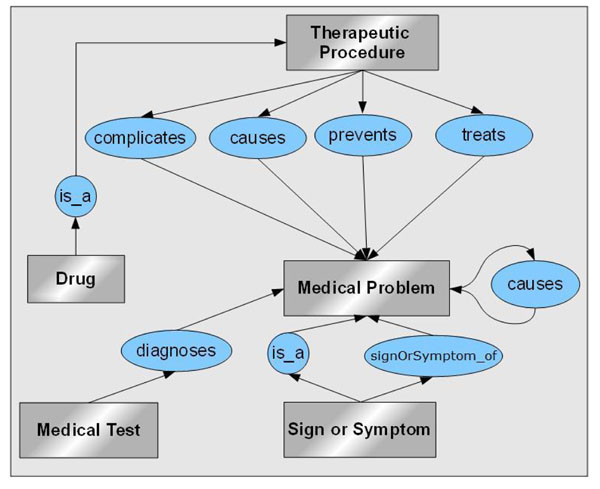
Excerpt of the relations model

Our approach is based on the use of linguistic patterns. For every couple of medical entities, we collect the possible relations between their semantic types in the UMLS Semantic Network (e.g. between the semantic types *Therapeutic or Preventive Procedure* and *Disease or Syndrome* there are five relations: *treats*, *prevents*, *complicates*, etc.). We construct patterns for each relation type (cf. the following section) and match them with the sentences in order to identify the correct relation. The relation extraction process relies on two criteria: (i) a degree of specialization associated to each pattern and (ii) an empirically-fixed order associated to each relation type which allows to order the patterns to be matched. We target six relation types: treats, prevents, causes, complicates, diagnoses and sign or symptom of (cf. Figure 1).

### Pattern construction

Semantic relations are not always expressed with explicit words such as *treat* or *prevent*. They are also frequently expressed with combined and complex expressions. Therefore, it is difficult to build patterns which can cover all relevant expressions. However, the use of patterns is one of the most effective methods for automatic information extraction from textual corpora if they are efficiently designed [13,16,17].

To build patterns for a target relation *R*, we used a corpus-based strategy akin to that of [11] and followers. We illustrate it with the *treats* relation. To apply this strategy we first need seed terms corresponding to pairs of concepts known to entertain the target relation *R*. To obtain such pairs, we extracted from the UMLS Metathesaurus all the couples of concepts connected by the relation *R*. For instance, for the *treats* Semantic Network relation, the Metathesaurus contains 45,145 *treatment-problem* pairs linked with the “may treat” Metathesaurus relation (e.g. *Diazoxide may treat Hypoglycemia*). We then need a corpus of texts where occurrences of both terms of each seed pair will be looked for. We build this corpus by querying the PubMed Central database [24] (PMC) of biomedical articles with focused queries. These queries try to identify articles that have high chances of containing the target relation between the two seed concepts. We aimed to optimize precision, therefore we applied the following principles.

• Since PMC, like PubMed, is indexed with MeSH headings, we restrict our set of seed concepts to those which can be expressed by a MeSH term.

• We impose a MeSH-based search mode to PMC by adding the /MH qualifier to the concepts.

• We also want these concepts to play an important role in the article. One way to specify this is to ask for them to be ‘major topics’ of the paper they index ([MAJR] field in PubMed or PMC; note that this implies /MH).

• Finally, the target relation should be present between the two concepts. MeSH and PMC provide a way to approximate a relation: some of the MeSH subheadings (e.g., *therapy or prevention and control*) can be taken as representing underspecified relations, where only one of the concepts is provided. For instance, *Rhinitis*, *Vasomotor/TH* can be seen as describing a *treats* relation (/TH) between some unspecified treatment and a *rhinitis*. Unfortunately, MeSH indexing does not allow the expression of full binary relations (i.e., linking two concepts), so we had to keep this approximation.

Queries are thus designed according to the following model: <*problem*>*/TH[MAJR] and* <*treatment*>/*MH*. They are submitted to PMC to obtain full-text articles on the required topics. This method should increase the chances of obtaining sentences where one of the reference relations occurs, and provides a large variety of expressions of the target relation.

The resulting corpus contains a set of medical articles in XML format. From each article we construct a text file by extracting relevant fields such as the title, the summary and the body (if they are available).

Then, we split every text into sentences using the segmentation model of the LingPipe project. We apply MetaMap on each sentence and keep the sentences which contain at least one couple of concepts (c1, c2) connected by the target relation *R* according to the Metathesaurus.

This semantic pre-analysis reduces the manual effort required for subsequent pattern construction, which allows us to enrich the patterns and to increase their number. The patterns constructed from these sentences consist in regular expressions taking into account the occurrence of medical entities at precise positions. Table 2 presents the number of patterns constructed for each relation type and some simplified examples of regular expressions. A similar process was performed to extract another different set of articles for our evaluation.

**Table 2:** Examples of relation patterns

Relation	Pattern number	Simplified examples
causes	28	* . . . E1 may trigger E2 . . .*
diagnoses	12	*E1 is the best test for (the diagnoses of)? E2*
treats	46	*. . . E1 was found to reduce E2 . . .*
prevents	13	*. . . E1 for prophylaxis against E2 . . .*

### Evaluation

To build an evaluation corpus, we queried PubMedCentral with MeSH queries (e.g. *Rhinitis, Vasomotor*/*th[MAJR] AND* (*Phenylephrine OR Scopolamine OR tetrahydrozoline OR Ipratropium Bromide)*). Then we chose a subset of 20 varied abstracts and articles (e.g. reviews, comparative studies).

**Figure 2:**
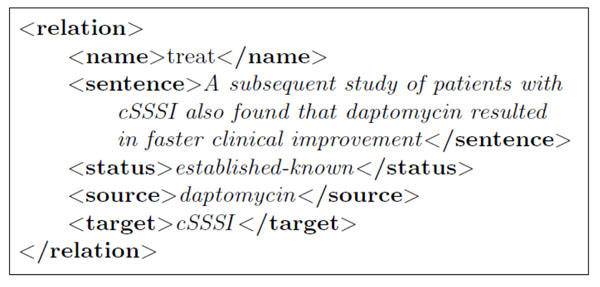
Example of manual annotations

We verified that no article of the evaluation corpus is used in the pattern construction process. The last stage of preparation was the manual annotation of medical entities and treatment relations in these 20 articles (total = 580 sentences). Figure 2 shows an example of an annotated sentence.

We use the standard measures of recall, precision and F-measure. However, correctness of named entity recognition depends both on the textual boundaries of the extracted entity and on the correctness of its associated category (semantic type). We apply a commonly used coefficient to boundary-only errors: they cost half a point and precision is calculated according to the following formula:(1)

• *C*: number of correct entities.

• *B*: number of entities with correct semantic type but incorrect boundaries.

• *T*: number of entities with wrong semantic types.

• *N*: total number of retrieved entities. (C + B + T = N)

The recall of named entity rceognition was not measured due to the difficulty of manually annotating all the medical entities in our corpus. For the relation extraction evaluation, recall is the number of correct treatment relations found divided by the total number of treatment relations. Precision is the number of correct treatment relations found divided by the number of treatment relations found.

## Results and discussion

In this section, we present the obtained results, the MeTAE platform and discuss some issues and features of the proposed approaches.

### Results

Table 3 shows the precision of medical entity recognition obtained by our entity extraction approach, called *LTS+MetaMap* (using MetaMap after text to sentence segmentation with LingPipe, sentence to noun phrase segmentation with Treetagger-chunker and Stoplist filtering), compared to the simple use of MetaMap. Entity type errors are denoted by *T*, boundary-only errors are denoted by *B* and precision is denoted by *P*. The *LTS+MetaMap* method led to a significant increase in the overall precision of medical entity recognition. Actually, LingPipe outperformed MetaMap in sentence segmentation on our test corpus. LingPipe found 580 correct sentences where MetaMap found 743 sentences containing boundary errors and some sentences were even cut in the middle of medical entities (often due to abbreviations). A qualitative study of the noun phrases extracted by MetaMap and Treetagger-chunker also shows that the latter produces less boundary errors.

**Table 3:** Medical entity extraction according to semantic types. Tr = T/N, type error rate; Br = B/N, boundary error rate; P = precision. All results are percentages.

	MetaMap			LTS+MetaMap		
	**Tr**	**Br**	**P**	**Tr**	**Br**	**P**

Disease Or Syndrome	9.09	52.27	64.77	9.81	26.48	76.94
Injury or poisoning	33.33	34.84	49.24	26.19	35.71	55.95
Neoplastic Process	29.03	6.45	67.74	37.5	12.50	56.25
Anatomical Abnormality	85.71	0.00	14.28	40.00	0.00	60.00
Cell or Molecular Dysfunction	66.66	25.00	20.83	44.44	44.44	27.79

Total	30.08	30.52	**54.62**	12.23	27.10	**74.21**

For the extraction of treatment relations, we obtained 60.46% recall, 75.72% precision and 67.23% F-measure. Other approaches similar to our work like [16] obtained 84% recall, 48.14% precision and 61.20% F-measure for the extraction of treatment relations. Semrep [20] obtained 54% recall, 84% precision and 68.21% F-measure on a set of predications including the treatment relationship (i.e. administrated to, manifestation of, treats). However, given the differences in corpora and in the nature of relations, these comparisons must be considered with caution.

### Annotation and exploration platform: MeTAE

**Figure 3:**
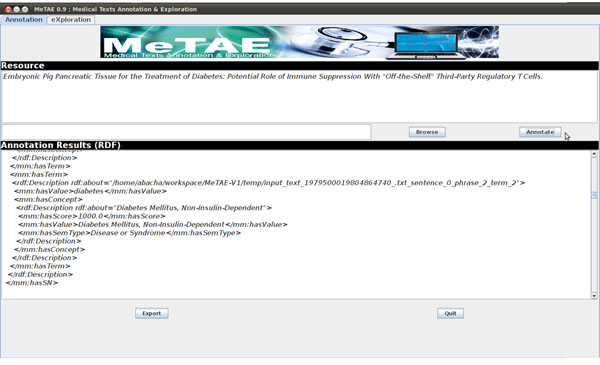
MeTAE: annotation interface

**Figure 4:**
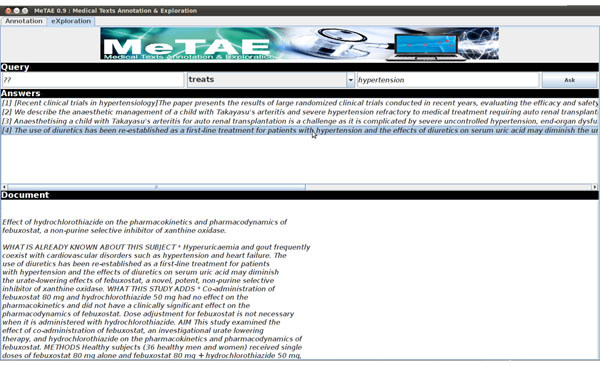
MeTAE: exploration interface

We implemented our approach in the MeTAE platform which allows to annotate medical texts or files and writes the annotations of medical entities and relations in RDF format in external supports (cf. Figure 3). MeTAE also allows to explore semantically the available annotations through a form-based interface. User queries are reformulated using the SPARQL language according to a domain ontology which defines the semantic types associated to medical entities and semantic relationships with their possible domains and ranges. Answers consist in sentences whose annotations conform to the user query together with their corresponding documents (cf. Figure 4).

### Discussion

Several semantic relation extraction approaches only address relation detection (e.g. find that a sentence contains the searched relation [16]). In the context of medical question-answering systems, we are not only interested in relation detection but also in the linked medical entities. We focus on searching <source,relation,target> triples such that the source and the target have known categories (semantic types) and such that the relation is valid w.r.t domain knowledge and w.r.t linguistic considerations (i.e. the sentence really states that the source treats the target). In this context, the same sentence may contain several triples <source,relation,target>.

A first analysis of the false positives shows that the main error causes are: (i) errors in the extraction of medical entities (ii) patterns of the treatment relation that also cover forms of expression of other relations and (iii) sentences that contain possible source and target entities without them being connected with the treatment relation.

Using external segmentation tools (LingPipe, Treetagger) brought improvements compared to the direct use of MetaMap. However, other segmentation tools exist and could display a different behavior. We performed a comparative study of a larger set of tools in a recent work [25].

It is interesting to note that our method brought new relation assertions between medical entities. For example, in the sentence: “Fosfomycin and amoxicillin-clavulanate appear to be effective for cystitis caused by susceptible isolates”, our system automatically extracted that fosfomycin (E1) and amoxicillin-clavulanate (E2) are two treatments for cystitis (E3) when no may treat relation is asserted between (E1,E3) resp. (E2,E3) in the UMLS Metathesaurus. The computation of the new assertions ratio is planned in a short term perspective.

A limitation to our approach is the fact that it will not always be the case that we have knowledge bases with semantic relationships between medical entities as a starting point. Also, the keyword and MeSH-qualifiers based method requires to have a specific qualifier for the target relation (here, TH for the treatment relation) to obtain a more focused corpus for pattern construction. If this is not the case, a decrease in the relevance of the obtained abstracts/texts may be expected.

A classic disadvantage of pattern-based methods is the expensive cost needed to obtain a good recall. Nevertheless, it is interesting to test and improve manual patterns to keep a good control on the extraction precision. Also, such methods can be integrated in hybrid extraction approaches to balance their qualities with that of statistical methods, as we did in recent work [26].

We obtained good results in precision and F-measure compared to other semantic relation extraction approaches. This meets our initial objective, which is to have a high precision in relation extraction in order to build efficient question-answering systems.

## Conclusions

In this paper, we presented a knowledge and linguistic-based approach for the extraction of medical entities and the semantic relations linking them. This approach is based on two main steps: (i) the recognition of medical entities with an enhanced use of MetaMap and (ii) the exploitation of linguistic patterns taking into account the semantic types of medical entities. The results obtained on a real test corpus show the effectiveness of our approach and its advantages for question-answering systems.

In short-term perspectives, we intend to study the false negatives in order to improve our patterns. We also intend to design a method which automatically extracts contextual information such as the status of the relation (e.g. hypothetical, established-known) and information about patients (e.g. gender, age).

## Competing interests

The authors declare that they have no competing interests.

## Authors contributions

ABA designed the proposed methods, built the relation patterns, implemented the platform, manually annotated the evaluation corpus and wrote a first version of the manuscript. PZ participated in the study design, proposed the MeSH-based method for corpus selection and contributed to the manuscript. Both authors read and approved the final manuscript.
